# Key Factors Influencing the Incidence of West Nile Virus in Burleigh County, North Dakota

**DOI:** 10.3390/ijerph15091928

**Published:** 2018-09-05

**Authors:** Hiroko Mori, Joshua Wu, Motomu Ibaraki, Franklin W. Schwartz

**Affiliations:** 1 Environmental Science Graduate Program, The Ohio State University, Columbus, OH 43210, USA; 2 College of Public Health, The Ohio State University, Columbus, OH 43210, USA; wu.1909@osu.edu; 3 School of Earth Sciences, The Ohio State University, Columbus, OH 43210, USA; ibaraki.1@osu.edu (M.I.); schwartz.11@osu.edu (F.W.S.)

**Keywords:** West Nile Virus, statistical modeling, North Dakota, *Culex tarsalis*, flooding

## Abstract

The city of Bismarck, North Dakota has one of the highest numbers of West Nile Virus (WNV) cases per population in the U.S. Although the city conducts extensive mosquito surveillance, the mosquito abundance alone may not fully explain the occurrence of WNV. Here, we developed models to predict mosquito abundance and the number of WNV cases, independently, by statistically analyzing the most important climate and virus transmission factors. An analysis with the mosquito model indicated that the mosquito numbers increase during a warm and humid summer or after a severely cold winter. In addition, river flooding decreased the mosquito numbers. The number of WNV cases was best predicted by including the virus transmission rate, the mosquito numbers, and the mosquito feeding pattern. This virus transmission rate is a function of temperature and increases significantly above 20 °C. The correlation coefficients (*r*) were 0.910 with the mosquito-population model and 0.620 with the disease case model. Our findings confirmed the conclusions of other work on the importance of climatic variables in controlling the mosquito numbers and contributed new insights into disease dynamics, especially in relation to extreme flooding. It also suggested a new prevention strategy of initiating insecticides not only based on mosquito numbers but also 10-day forecasts of unusually hot weather.

## 1. Introduction

The West Nile Virus (WNV) is an arbovirus, which operates in a natural enzootic cycle between mosquito vectors and avian hosts [1]. Currently, no vaccine or treatment has been developed for humans [2]. The absence of medical remedies for WNV points to the need for strong mosquito control programs for protection against the virus. Another important reason for focusing on WNV infections in humans is a large number of unprecedented outbreaks in the North-Western hemisphere. After the initial introduction of the virus in 1999, it spread across North America in just a few years. Since then, the virus has been responsible for more than 40,000 cases among humans within the U.S. [3]. Its broad geographic distribution and ability to adapt to new habitats have raised concerns about the evident difficulties in controlling disease outbreaks.

Several statistical approaches have been developed to discover and investigate the critical factors that control the severity of WNV outbreaks. Studies have set out to model how changes in environmental and other factors contribute to the dissemination of the virus [4,5,6,7,8]. Given the close association of the disease with mosquitoes, a common approach to understanding its transmission has involved modeling the population dynamics of mosquitoes in terms of different climatic factors [6,7,9,10,11] or the mosquito distributions by using land use [12]. For example, temperature is closely linked to mosquito birth and mortality rates and the length of the gonotrophic cycle [13]. Rain is also considered important in providing the small water bodies necessary for certain mosquitoes to reproduce. However, relationships can be complex because not all mosquitoes have the same water requirements and higher rainfalls can end up washing away larva or eggs [4]. The land use can affect the mosquito species distributions because each species has its preferential habitat for breeding eggs.

One reason for focusing on modeling mosquito populations is the inference that the disease risk increases with higher numbers of mosquitoes. However, information on mosquito numbers alone may not be sufficiently predictive of WNV cases [7,14]. Extensive WNV activity can be evident during periods with low mosquito abundance [7,14]. To account for this complexity, others, for example, Chung et al. [7], and Kilpatrick and Pape [8] used the detected numbers of infected mosquitoes (e.g., vector index) as a basis for correlations with numbers of human disease cases. The vector index turns out to be highly correlated with human disease cases, but not necessarily associated with the abundance of mosquitoes. Kilpatrick and Pape [8] developed a model using this index to predict outbreaks of WNV several weeks in advance. Despite its effectiveness for forecasting virus activity, not every local health department has the ability to conduct WNV testing due to funding limitations or lack of capacity for testing [15]. Thus, limitations often exist in obtaining continuous surveillance data on the numbers of infected mosquitoes. Therefore, an alternative approach to predict the WNV cases could be helpful in areas where mosquito surveillance information with respect to the vector index is either limited or unavailable.

Other studies have suggested that besides the vector index, virus transmission factors play an important role in virus dissemination [16,17,18,19,20,21,22,23,24,25]. Kilpatrick et al. [23] found that high temperatures accelerate the infectiousness of mosquitoes due to an increase in viral replication rates. Increasing temperatures also contribute to an increase in the frequency of mosquito bites. This increased activity leads to greater numbers of WNV infections [17]. Another important factor leading to virus transmission is the seasonal changes in mosquito feeding behaviors [19]. Their study showed a shift in preferential hosts from birds to humans in late summer. Such host switching behavior amplifies the numbers of WNV infections. While these factors are likely influential in controlling virus transmission, their influence has not been fully quantified in terms of the numbers of reported human disease cases. Further analysis of these factors in combination with information on mosquito abundance could lead to the capability to predict the numbers of infected humans. We envision such an approach to be useful in providing strategies for the prevention and control of WNV.

Beyond a specific focus on factors that influence virus transmission, other studies have found success in directly modeling the numbers of infected individuals through direct correlations with climatic factors [7,26]. For example, Chung et al. [7] found that a warmer winter can lead to an increase in WNV transmission the following summer. Mild winters contribute to the overwintering of a larger number of infected mosquitoes, which in turn yields a greater number of infected mosquitoes in early spring. In addition, Stililanakis et al. [26] found that temperature was positively correlated with the numbers of disease cases, while humidity and soil water content were negatively associated. Warmer temperatures lead to faster larval development, a reduction in the incubation period of infected mosquitoes, and an earlier initial migration of bird hosts, which can transport the virus. A scarcity in water increases the density of hosts and vectors around open water reservoirs and facilitates interactions which result in greater virus transmission. This finding suggests that warmer temperatures associated with drought conditions will increase the risk of outbreaks. The works by Chung et al. [7] and Stililanakis et al. [26] are important for identifying the direct association of the numbers of human disease cases with climatic factors. Conceptually, these climatic factors influence both mosquito behavior and the transmission dynamics of WNV. It would be beneficial to differentiate the combined effects of factors in order to explicitly separate factors responsible for the changes in mosquito abundances from those contributing to the disease cases.

In this study, we investigate the population dynamics of female *Culex tarsalis*, a mosquito which is a key vector for WNV transmission [10,27,28]. Our purpose here is to determine the factors that contribute to the WNV outbreaks and ultimately to provide strategies for controlling the dissemination of the virus. The study has two objectives. First, we develop a predictive statistical model to evaluate factors that amplify mosquito numbers. The second objective involves the development of an alternative statistical approach for predicting the numbers of disease cases using raw mosquito numbers in combination with transmission factors. This overall approach is an alternative to methods based on the vector index and will be useful for those counties that conduct limited mosquito surveillance. This study will lead to an improved understanding of the most important transmission factors associated with virus dissemination and the disease ecology of WNV, more specifically, conditions favoring the proliferation of WNV vectors. Eventually, such knowledge could ultimately lead to a reduction in disease outbreaks.

## 2. Materials and Methods

### 2.1. Study Area

**Figure 1:**
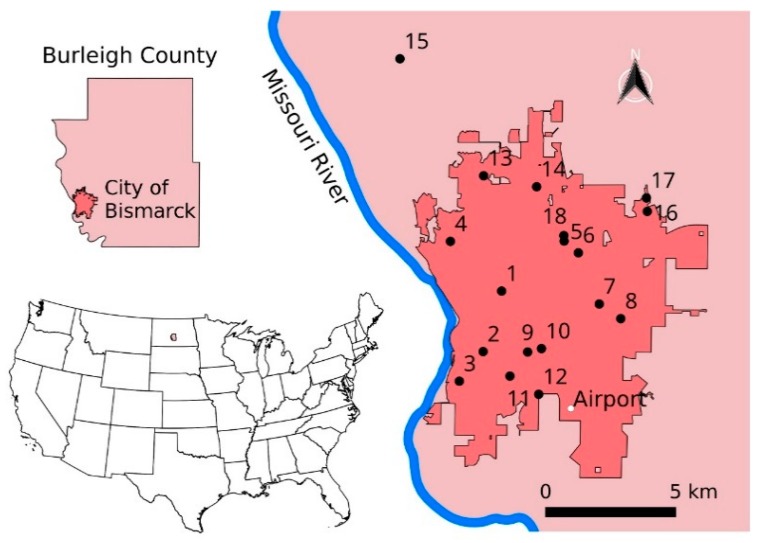
Maps showing the human disease surveillance area and the locations of mosquito traps. The numbers indicate the locations of mosquito traps. Location 15 is located outside of the city of Bismarck although it is still within the metropolitan area. The map was created using multiple shapefiles obtained from the United States Census Bureau [29] and the North Dakota GIS Hub Data Portal [30,31,32,33,34].

The study area is located in the City of Bismarck (Burleigh County) in North Dakota (ND) along the Missouri River (Figure 1).

The city covers an area of ~81 km^2^. It was selected because it has among the highest incidences of WNV per population size in the U.S. [3] and has available weekly data on mosquito numbers and disease cases across a number of years. This area is characterized by long, cold winters with snowfalls and hot, dry summers with sporadic rainstorms. The annual temperature ranges from 30 °C to −15 °C with an annual average precipitation of 487.9 mm. [35,36]. The area is predominantly agricultural with farmland arrayed among thousands of perennial, pothole lakes [35].

### 2.2. Data on Mosquito Numbers and West Nile Virus Cases

Data on weekly mosquito numbers were available for 2007–2016 from the Environmental Health Division for the City of Bismarck. The New Jersey mosquito traps employed by the city capture a broad spectrum of mosquito species [37]. Each year for 15 weeks (end of May to early September), traps were maintained at ten suitable sites in Bismarck. On average, one trap monitors about eight km^2^ of the city. The trap locations were occasionally changed from year to year (Table S1 in Supplementary Materials). Thus, mosquito trap data came from 18 sites from 2007 to 2016 (Figure 1). On average, a trap was operated at a single location for 5.0 years with a standard deviation of 2.6 years. Traps were operated every night from dusk to dawn using installed programmable timers. At the end of each week, the trapped mosquitoes were collected. Mosquitoes were counted and identified based on species and gender. Our analyses focused on the numbers of female *Culex tarsalis* mosquitoes because they are the most common vector for WNV in ND [10,27,28]. No spraying with insecticides or larvicides was conducted at the trap sites. Spraying might have occurred in the neighborhood of the traps, but their impacts might be minimal [38] and not considered in our analysis.

Weekly data on the number of human disease cases of WNV for Burleigh County were obtained from 2007 to 2016. In this study, human disease cases include both neuroinvasive and non-neuroinvasive presentations. WNV cases mainly occurred from early June to late September. Unlike the mosquito data, the weekly numbers of disease cases were tabulated year-round.

**Figure 2:**
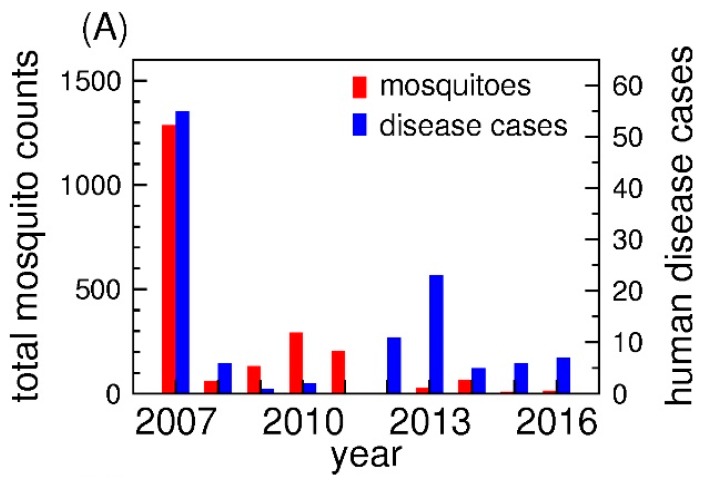

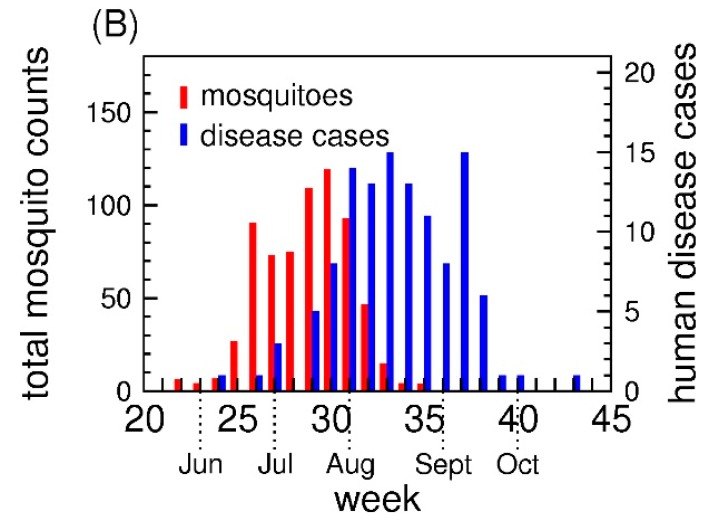
The incidence of human disease cases of West Nile Virus in Burleigh County and mosquito numbers in Bismarck. Panel (**A**) shows the averaged yearly total of *Culex tarsalis* mosquitoes per trap and the yearly total of human disease cases. The mosquito numbers represent the total mosquito numbers from all traps divided by the total number of traps operated each year. Total number of human disease cases are the numbers of patients diagnosed with WNV each year; Panel (**B**) displays the averaged weekly total numbers for *Culex tarsalis* mosquitoes and human disease cases from 2007 to 2016. The mosquito counts were calculated by aggregating trap data for the same week of the year and dividing this sum by the total number of traps. The disease cases were aggregated for the same weeks of the year. The red bars indicate the total number of mosquitoes counted while the blue bars are the total number of human disease cases.

For the statistical modeling, mosquito numbers were averaged weekly across all trap locations. Those locations where no data were collected in a given week were excluded. We also excluded data from traps that were considered by the Public Health Department to have malfunctioned. Figure 2A shows the variation in the annual mosquito numbers and human disease cases through the study period. Mosquito numbers were calculated each year as the total of all *Culex tarsalis* mosquitoes collected from all traps divided by the number of traps operated (potentially 10). Total human disease cases were the number of patients who were diagnosed for WNV in Burleigh County each year. For the seasonality comparison (Figure 2B), mosquito numbers were aggregated for the same week of the year and averaged over the total number of traps. For human disease cases, we aggregated the disease cases for the same week of the year.

**Figure 3:**
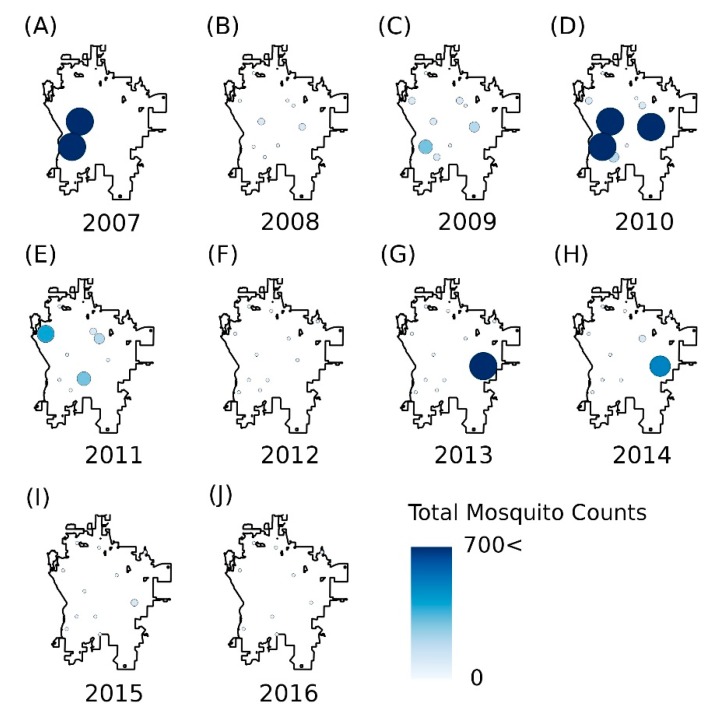
Maps showing the yearly total of mosquitoes from 2007 to 2016 (**A**–**J**). The area of the circle and color show the mosquito numbers (count). It is evident from the annual maps that the numbers and locations of mosquito fluctuate from year to year.

In addition to the aggregated data on mosquito numbers, we also summarized annual data for each of the traps. The yearly data on mosquito abundances for each trap facilitated the preparation of mosquito distribution maps for 2007–2016 (Figure 3). The maps were constructed using the inverse-distance-weighting plugin in QGIS 2.18 software [39].

### 2.3. Climatological Factors Considered for the Mosquito Model

**Figure 4:**
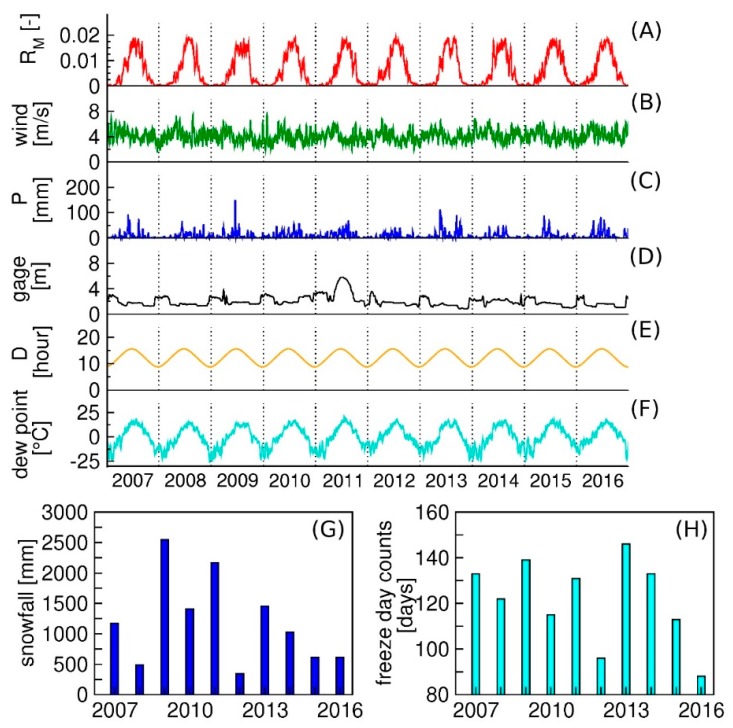
Time series for the ecological factors tested to predict mosquito numbers between 2007 and 2016. Panel (**A**) shows reproduction rate of mosquitoes (*R_M_*); Panel (**B**) wind velocity; Panel (**C**) precipitation (*P*); Panel (**D**) gage height of Missouri river; Panel (**E**) day length (*D*); Panel (**F**) dew point; Panel (**G**) snowfall; and Panel (**H**) the numbers of freeze days. Variables shown in panels (**A**–**F**) are described on a weekly basis. Variables in panel (**G**–**H**) have a yearly basis.

We examined eight factors to elucidate their potential role in controlling mosquito abundances in Burleigh County, ND. The factors examined on a weekly basis were mosquito reproduction rate, cumulative precipitation, gage height for the Missouri River, day length, dew point, and wind velocity. Those factors with a yearly basis were snowfall and the number of freeze days (Figure 4). These or similar variables have formed the basis of other statistical models [6,14,40,41,42]. What is unique about our approach is that we calculated the mosquito reproduction rate using temperature to consider the non-linear dynamics of the mosquito life cycle instead of using raw temperature values. Including the gage height of the nearby river is also a novel approach that incorporates effects of over-bank flooding on mosquito numbers. Over-bank flooding occurs when river water overflows and inundates the floodplains along the river. The inundations of floodplains can either form or wash away suitable water bodies for mosquitoes to breed. Using these variables, we developed a statistical model to predict weekly mosquito numbers for Bismarck.

The mosquito reproduction rate (*R_M_*) (per day) is a function of temperature and is calculated using mean temperature (°C) (Appendix S1 in Supplementary Materials) obtained from a weather station at Bismarck (Figure 1). *R_M_* was calculated by subtracting the mortality rate from the birth rate of mosquitoes and then averaged on a weekly basis. The duration of high *R_M_* (temperature-driven) for adult mosquitoes was longer in 2008, 2012, and 2013, which suggests that the mosquito life cycle was faster in these years as compared to others. Not surprisingly, the reproduction rates spiked during the warm summer months and were zero in winter. This latter result implies that mosquitoes are in the state of diapause for overwintering [43]. The pattern of seasonal variability was consistent from 2007 to 2016 (Figure 4A).

Wind velocity (m/s) was also included as a model variable. It has an influence on the flight range of mosquitoes and often large wind velocities restrict the appetitive flights of mosquitoes [44]. Furthermore, because mosquitoes detect CO_2_ in their search for blood meals, wind velocity can be important [44]. Wind velocity data were obtained from the weather station at Bismarck Municipal Airport [45].

Precipitation (mm) was considered as a factor because rainfall has a substantial impact on the availability of mosquito breeding sites and the possibility of extreme events that destroy mosquito eggs and larva [40,46]. Precipitation might also be included as a variable within the functions describing mosquito reproduction rate. However, we included precipitation as an independent variable in the model. As is typical of the northern Great Plains, precipitation varies considerably from one year to the next (Figure 4C). For example, 2012 and 2015 were relatively dry years, while 2007–2009, and 2011 were relatively wet. Daily values of mean temperature and precipitation are available from the weather station at Bismarck Municipal Airport [45].

The gage height (m) for the Missouri River is an indicator of river flow conditions that range from typical summer low flows to over-bank flooding. Under low flow conditions, stagnant water on the floodplain provides mosquito breeding sites. Overbank flows associated with higher stages, lead to the disruption of these sites. Given the potential for flooding on the Missouri River, we included gage height lagged one year to account for this effect. The stage of the Missouri River, as measured by the U.S. Geological Survey gage at Bismarck, is regulated upstream by the Garrison Dam. River flows are typically highest in winter (Figure 4D) as water is released to free up reservoir storage for expected spring flooding. Low flows typically occur in summer through fall and decline slowly through the warm season. The obvious exception is 2011, where the combination of record snows in the Rocky Mountains and near-record rainfalls in eastern Montana resulted in severe flooding, eventually leading to emergency releases from the Garrison Dam. The high stages recorded in 2011 (Figure 4D) reflect months of downstream flooding that impacted Bismarck. Because the flooding was due to events much further upstream, this excess spring precipitation is not reflected in recorded precipitation at Bismarck (Figure 4C). Daily mean gage heights for the Missouri River at Bismarck were obtained from the U.S. Geological Survey National Water Information System (Station 06342500).

The behavior in mosquito-population numbers is affected by day length (hours of daylight (hour)) [47]. The day length changes seasonally (Figure 4E) and mosquitoes start to diapause once day lengths begin to shorten [43]. This response by the mosquitoes suggests that the day length could determine the end of the mosquito season. Diapause is a dormant stage for mosquitoes, which occurs with *Culex* mosquitoes in the form of adult.

Weekly average dew point temperature (°C) was included in the model. The dew point lets us test whether moisture in the air would affect the mosquito population dynamics. The dew point typically is relatively high in late summer. The summer of 2013 was an exception with a marked increase evident in early summer (Figure 4F). Dew point data were retrieved from the PRISM (parameter-elevation regressions on independent slopes model) data product [48].

Total snowfall amounts (mm) and numbers of freeze days (days) (temperature below 0 °C) were tabulated between October and April (Figure 4G). We included these variables in the model because in this climatic setting, the accumulated snowfall is a reasonable proxy for the quantity of snowmelt runoff in spring, which potentially could increase the number of waterbodies available as oviposition sites. In addition, the numbers of freeze days could affect the population of overwintering mosquitoes. The snowfall and freeze day data suggest that 2012 featured a mild, dry winter, while winters from 2009 through 2011 were cold and wet. Winter in 2008 was relatively cold but dry (Figure 4G,H). Total snowfall amounts and numbers of freeze days were calculated from climate data provided by the weather station at Bismarck Municipal Airport [45].

### 2.4. Transmission Factors Considered for the Model of Human Disease Cases

**Figure 5:**
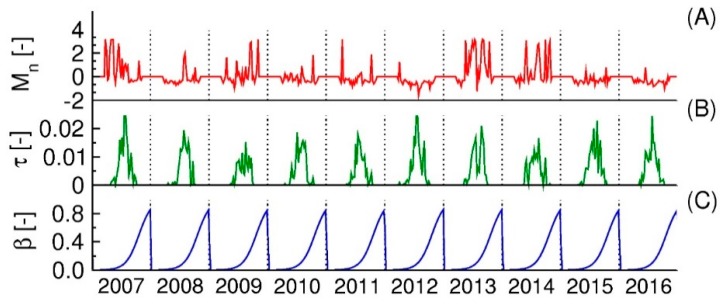
Time series for key variables used to predict the dynamic of human disease cases from 2007 to 2016. The three panels display time series of transmission factors used for the model of human disease cases. Panel (**A**) shows normalized mosquito numbers (*M_n_*); Panel (**B**) transmission rate of the virus (*τ*) of mosquitoes; and Panel (**C**) feeding patterns of a mosquito on humans (*β*). All the variables have a weekly basis.

In this section, we describe variables potentially important in determining the numbers of humans infected by WNV. Literature [13,16,19,23,49,50] guided the choice of relevant factors. Regardless of their strong influence on disease transmission, it is not known how the variability in these factors affects the number of WNV cases. Here, we considered three transmission factors: (i) normalized weekly total in mosquito numbers (–); (ii) weekly averaged virus transmission rate (–); and (iii) feeding patterns of mosquitoes (–) (Figure 5).

Weekly mosquito numbers obtained from the mosquito model were also included as a variable in the statistical model. Here, we normalized the weekly averaged number of mosquitoes (*M_n_*) (–) as follows,

(1) $$M_{n}=\frac{M-M_{ave}}{M_{std}}$$

where *M* is weekly mosquito numbers obtained from the mosquito model, *M_ave_* is weekly averaged of mosquito numbers, and *M_std_* is a standard deviation in weekly mosquito numbers of each week between 2007 and 2015. We applied the normalized weekly mosquito numbers of the city to predict the disease cases for all of Burleigh County. The mosquito numbers were high in 2007, 2009, 2011, 2013, and 2014. In 2012, the normalized numbers remained low throughout the year (Figure 5A).

To measure how likely mosquitoes are to transmit WNV to humans, the virus transmission rate (*τ*) was calculated by multiplying the rate of virus development (*υ*) (–) and the mosquito biting rate (*ε*) (bite counts/week). The υ is the inverse of the extrinsic incubation period of mosquitoes. Reisen, Fang & Martinez [50] incubated infected mosquitoes at constant temperatures from 14 to 30 °C, with incubation periods measured at each incubation temperature. The incubation periods were plotted as a function of temperature. By fitting a regression line to the plots, they found no virus development below 14.3 °C. Using this regression line, we calculated the weekly averaged rate of virus development in mosquitoes.

Note that *ε* is also a function of temperature. The function used here comes from the laboratory study of Rubel et al. [13] and quantifies how many times a mosquito bites hosts per day. The equations for the rate of virus development and the biting rate are presented in Appendix S1 in Supplementary Materials. The inter-annual variability in the transmission rate of WNV is due primarily to the temperature variation. Values of *τ* were high during 2007, 2012, and 2013. The lowest values were observed in 2009 and 2014 (Figure 5B).

Information on the feeding patterns of mosquitoes came from a study by Kilpatrick et al. [19]. They used molecular biological assays to identify the sources of blood meals for wild *Culex* mosquito between May to September in Maryland and Washington D.C. The proportion of blood meals from humans increased gradually from spring to late summer (Figure 5C). This shift in feeding preference explains the increase in human infections in later summer (Figure 2B). The equations to derive the seasonal fraction of human blood meals (*β*) are listed in Appendix S1 in Supplementary Materials.

### 2.5. Statistical Analyses and Model Evaluation

Generalized linear models (GLMs) were employed to model the weekly mosquito numbers and human disease cases. Unlike classical linear regression models, GLMs can accommodate dependent variables with non-normal distribution and provide for nonlinear relationships between dependent and predictor variables by changing its link functions [51,52,53]. Multiple generalized linear models (GLMs) were compared to examine the most plausible statistical model to predict the number of mosquitoes and human disease cases. For comparison, we used the Vuong test to select the best model among different candidates [54,55]. A negative binomial regression model was chosen to model weekly mosquito numbers [55,56]. A zero-inflated negative binomial regression was selected to model weekly human disease cases because it can handle the many zero values occurring in the data (i.e., ~80%). Additional information concerning the regression model is presented in Appendix S2 in Supplementary Materials.

Climatological factors were used to predict the mosquito numbers, and transmission factors were employed to estimate human disease cases. To assess time lag effects in weekly variables, we built lags of 0, 1, and 2 weeks into each weekly variable. The time lag producing the lowest Akaike’s information criterion (AIC) for each variable was included in the model. We also checked the variance inflation factor (VIF) to test the collinearity between variables [42].

Clearly, some of the variables in the models are entirely theoretical. For example, mosquito reproduction rate and virus transmission rate are empirical equations based on measurements made in a laboratory under constant temperature conditions [13,57]. Although temperatures do indeed vary daily at Bismarck, weekly mean temperatures were used to estimate those variables in this study. In order to examine the effects of variability in these temperature functions, we calculated a confidence interval (CI) of 95% for each temperature functions and conducted a sensitivity analysis. The calibration of these variables for the actual field conditions in the area may improve the fitness of the model in future studies.

Regression analysis was performed for all possible combinations of variables. This kind of analysis provides a quantitative basis for identifying key factors important in determining the mosquito numbers and human disease cases. The best combination of variables was identified based on AICc (second-order Akaike’s information criterion). AICc is a better method, as compared to AIC when the sample size is small [14]. As with AIC, the smallest AICc value identifies the best model with the fewest variable combinations.

The available nine years of data between 2007 and 2015 for mosquitoes and human WNV cases were used for statistical modeling. As mentioned, the mosquito model was fitted to 15 weeks of data that excludes cold months. The number of human disease cases was fitted to 19 weeks of data between May and October, a critical period for infections.

A leave-one-out cross-validation was performed to evaluate the performance of the models, following approaches in other studies [6,14,58]. One year of data on mosquito and case numbers were set aside as validation data and the remaining seven years were used in creating best-fit models. These fitted models were used to predict the mosquito and case numbers for the excluded years and the root mean square error (RMSE) was calculated to compare the predicted values from the fitted model with the excluded data. This cross-validation analysis was repeated nine times. The RMSE was also calculated for the resulting statistical models, which were fitted with all nine years of data and compared to the RMSE of cross-validation results to evaluate the accuracy of the model. In addition, the mosquito and disease case data for 2016 were used to investigate the prediction accuracy of the models as out-of-sample data. Pearson’s correlation coefficient was estimated to quantify how well the regression models predicted the observed weekly mosquito numbers and human disease cases [59]. All statistical analyses were conducted using RStudio 1.0.136 [60] built on R version 3.2.3 [61].

## 3. Results

### 3.1. The Number of Human Disease Cases and Mosquitoes

Mosquito numbers were highest in 2007, and lowest in 2012, followed by 2015 (Figure 2A). Aggregated weekly mosquito numbers were largest from late July to the beginning of August (Figure 2B). The largest number of human WNV cases was reported in 2007. In 2011, there were no human cases (Figure 2A). The seasonal variability in the number of disease cases was particularly evident in August through early September when it exhibited the highest incidence (Figure 2B). There were no reported cases of WNV during the colder months from November through May.

In general, yearly data on mosquito abundance did not correlate strongly with yearly data on human disease cases (Figure 2A). For example, the relatively large number of cases observed in 2012 coincided with relatively small numbers of *Culex tarsalis* mosquitoes. Also, larger mosquito numbers in 2010 were associated with only a few WNV cases. Comparisons of weekly cases with numbers of *Culex tarsalis* mosquitoes (Figure 2B) showed that the peak in WNV cases lagged behind the peak in mosquito numbers by several weeks or more. Mosquito abundance usually peaked in late July while the numbers of human disease cases peaked in August and September.

The year-to-year variation in mosquito abundances was analyzed for those traps that were sampled continuously from 2007 to 2016. We determined that mosquito numbers at locations 1 and 4 (Figure 1) were strongly correlated (*r* = 0.65). Similarities in mosquito fluctuations were likely because both traps were located adjacent to the Missouri River. At other sampling locations, for example location 14, mosquito numbers were poorly correlated with those at locations 1 and 4 (*r* = 0.28). This trap was located to the northeast, further away from the river. Mosquito numbers recorded for this location were also smaller than at the two locations closer to the river. These results suggest considerable heterogeneity in the distribution of mosquitoes.

Variability in the distribution of mosquitoes is more evident from spatial analysis of yearly mosquito numbers for Bismarck (Figure 3). In the years after 2011, trap data indicate a significant decline in mosquito numbers on the western side of the city closer to the Missouri River (Figure 3). The Missouri River experienced significant over-bank flooding in 2011, which might have contributed to the reduction in mosquito numbers. Favorable mosquito habitats along the Missouri River were likely disrupted with eggs flushed away by the high flows. This extreme event likely produced the variability in mosquito numbers from the eastern to the western ends of the city.

### 3.2. Identification of Key Factors Affecting Mosquito Abundances

A negative binomial regression model was developed to describe mosquito numbers in Bismarck. Based on the VIF test, none of the model variables show significant collinearity (VIF < 4.0). We chose the best model providing the smallest AICc using all possible combinations of variables (Table S2 in Supplementary Materials). Six key variables were determined from the best-fit model: (i) weekly reproduction rate of mosquitoes at lag zero-week; (ii) weekly dewpoint at lag one-week; (iii) the gage height of the Missouri River with a lag of zero-weeks; (iv) the gage height with a lag of one year; (v) the daytime length at lag two-weeks; and (vi) the total number of previous winter freeze days (Table 1).

The closest competing model (Table S2 in Supplementary Materials) included the same parameters except for gage height with a lag of two-weeks. As shown by the tabulation in Table 1, the abundance of *Culex tarsalis* mosquito was highly associated with ecological variables at lag zero and lag two weeks. The two-week lags in the variables in relation to mosquito abundance may have been associated with the life cycle of mosquitoes. Mosquitoes require 10–14 days to develop from egg rafts to adults, which shifts the timing in the behavior of the population relative to the variables.

### 3.3. Identification of Key Factors Affecting Human Disease Cases

The factors affecting the numbers of human disease cases were investigated using a zero-inflated negative binomial model. Table 2 shows the collection of variables providing the smallest AICc in the model of disease cases. The best-fit model for cases of WNV in humans included only three variables: (i) normalized weekly numbers of *Culex tarsalis* mosquitoes at lag two-weeks; (ii) weekly transmission rate of the virus (temperature-driven) at lag two weeks; and (iii) weekly fraction of human blood meal at lag two-weeks (Table 2). The competing model included two variables, the transmission rate of the virus at lag two-weeks and the weekly fraction of human blood meal of lag two-weeks (Table S3 in Supplementary Materials).

### 3.4. Model Validation for Mosquito Numbers and Human Disease Cases

A leave-one-out cross-validation was conducted to examine model performance. The RMSE was calculated for each excluded year. For both the mosquito and human disease model, the RMSE obtained from cross-validation were comparable to the RMSE calculated from the model fitted with full nine years of data (Tables S4 and S5 in Supplementary Materials). One exception was that the mosquito model showed a relatively large cross-validated RMSE value for 2007 (RMSE = 149.2), 2012 (RMSE = 5412), and 2013 (RMSE = 402.0) as compared to the model fitted using nine years of data. 

Out-of-sample data for 2016 were available to test the prediction accuracy of the model. Calculated values of RMSE and the Pearson’s correlation coefficient showed that the model successfully captured the variability in mosquito numbers (RMSE = 8.96, *r* = 0.910). The model of human disease cases also performed well in predicting the 2016 incidence of WNV cases (RMSE = 0.541, *r* = 0.620). 

**Figure 6:**
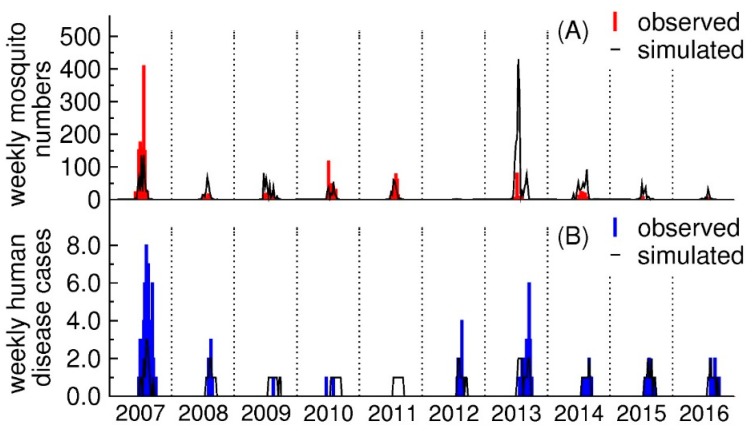
Comparisons between observed and simulated mosquito numbers and human disease cases. Panel (**A**) shows weekly numbers of female *Culex tarsalis* mosquitoes and Panel (**B**) shows the numbers of weekly human disease cases. The observed mosquito numbers are the averaged data from all traps.

Figure 6A,B provide a comparison of observed and simulated mosquito numbers and human disease cases based on the best-fit models. A visual comparison of results shown in Figure 6A indicates that the model of mosquito case numbers tracked the observed numbers through most years and particularly the years with small mosquito numbers and late in the annual mosquito seasons (i.e., September). The mosquito model did not capture the peak effectively for some weeks in 2007 (RMSE = 108, *r* = 0.760) and 2013 (RMSE = 182, *r* = 0.175). The model of WNV case numbers did well in predicting the summer spikes in numbers for 2012 (RMSE = 0.795, *r* = 0.630), and 2013 (RMSE = 1.34, *r* = 0.544). However, the model overestimated the number of actual cases in 2011 (RMSE = 0.659, *r* had no value because the number of human disease cases were zero in 2011) and underestimated the peak in 2007 (RMSE = 2.84, *r* = 0.789).

The sensitivity analysis on the mosquito reproduction rate (temperature-driven) on the mosquito model showed significant changes in the prediction of mosquito numbers. The mosquito reproduction rate is calculated by subtracting the mosquito mortality rate from the mosquito birth rate. The birth and mortality rates are functions of temperature and these functions have lower and upper bound of the 95% CI. When the lower bound of the 95% CI was used to calculate the mosquito reproduction rate, the mosquito model predicted a drastic increase in the mosquito numbers (Figure S1 in Supplementary Materials). This result occurs because the mosquito mortality rate approaches zero at the lower bound of the 95% CI; therefore, the reproduction rate becomes large. When the reproduction rate was adjusted to the upper bound of the 95% CI, the model predicted smaller mosquito numbers (Figure S1 in Supplementary Materials).

For the model of human disease cases, the temperature was used to calculate the virus transmission rate. Recall that the virus transmission rate is the product of the mosquito biting rate (temperature-driven) and the virus replication rate (temperature-driven). According to Figure S1 in Supplementary Materials, the virus transmission rate is a relatively insensitive variable in predicting human disease cases. Figure S1 showed a slight increase in human disease cases when the upper bound of the 95% CI of transmission rate was used, while the slight decrease in human disease cases was observed with the lower bound 95% CI.

## 4. Discussion

This study identified key factors that contributed to variability in the mosquito numbers and WNV cases in Bismarck, North Dakota. Our approach is based on separate statistical models for mosquito numbers and human disease cases. This has the advantage of being able to examine mosquito abundances independently of factors related to disease transmission. This capability allows us to analyze issues directly associated with disease outbreaks. Other studies commonly considered relationships between climatic factors and mosquito abundances [7,9,10,11] or climatic factors and the human disease cases [7,26].

We found a strong correlation between measured and simulated mosquito numbers in the statistical model with a relatively small number of variables (Table 1). Mosquito reproduction rate (temperature-driven) turn out to be a key variable for modeling mosquito numbers. The sensitivity analysis also showed that small changes in mosquito reproduction rate could alter prediction of mosquito numbers. Overall, the temperature is an important factor because it accelerates the mosquito life and gonotrophic cycles [57].

Dew point was also an important factor and positively correlated with mosquito numbers. Thus, the weather associated with high dew points may create a risk for larger numbers of mosquitoes. We also found that day length and number of days below 0 °C (freeze days) were positively correlated with mosquito abundance. The day length result suggests that seasonality may affect mosquito activity. In addition, our model showed that cold winter weather might increase the risk of a serious mosquito outbreak during the following spring and summer.

The correlation between mosquito numbers and gage height also suggest that flood events might negatively affect mosquito abundances. For Bismarck, the effect of flooding was examined by including the Missouri River gage height at lag one year in the statistical model. While wet conditions from flooding [53,62] may lead to higher numbers of mosquitoes, the extreme flooding event along the Missouri River in 2011 likely contributed to reduced mosquito populations in subsequent years. The other studies [29,31,38] also suggested that the dynamics of rivers and the effects of hydrological conditions can influence the mosquito abundance and the risk for WNV infections in humans. This gage height result implies that riparian lands along the Missouri River near Bismarck could provide important mosquito habitat and a target for amelioration strategies. Interestingly, this flooding event was not caused by local precipitation during summer but by the snowmelt runoff much further upstream. This finding highlights the importance of considering hydrological processes more explicitly in order to understand the behavior of mosquito populations.

Our modeling also finds a strong correlation between numbers of WNV cases and transmission factors. The key variables include the normalized weekly numbers of *Culex tarsalis* mosquitoes, the transmission rate of the virus (temperature-driven), and the feeding pattern of mosquitoes on humans. The statistical analysis shows mosquito numbers to be an important factor in predicting WNV cases. This result is in contrast to our findings with the yearly comparison between mosquito numbers and human disease cases (Figure 2). Differences in the strength of correlations are likely due to differences in temporal scales of averaging on a yearly versus weekly basis. Analysis with a fine temporal scale can yield a refined understanding of vector-host dynamics. Furthermore, the transmission rate of the virus (temperature-driven) was the only statistically significant variable in the log-linear part of the zero-inflated negative binomial regression model while in the logit part, all variables were statistically significant. The log-linear part expresses the zero count of the model while the logit-part gives counts greater than zero. This suggests that when the numbers of disease cases are above zero, we need to account for the influence of multiple factors to estimate the numbers of disease cases. Therefore, multivariate regression analysis can be useful in establishing the complex effects of multiple variables in controlling the number of disease cases.

Mosquito trap data from 49 different sites scattered across ND were used to examine the variability in yearly mosquito numbers between 2007 and 2015 (Appendix S3 in Supplementary Materials). The purpose of this analysis was to test the proposition that low mosquito abundances in 2012 observed for Bismarck was not due simply to errors in mosquito collection. These data confirmed that mosquito numbers were small at all 49 sites (Figure S2 in Supplementary Materials). Furthermore, the nine-year time series in mosquito numbers for Bismarck was consistent with other regions in ND. This analysis indicates that mosquito data obtained from Bismarck is not anomalous. Some studies [7,14] also indicated that there are years with small mosquito numbers associated with relatively large numbers of human disease cases. This association is what was observed for Bismarck in 2012. In other words, the 2012 observation is not likely to have resulted from limitations in the data.

What is significant about our disease case modeling is that the transmission rate of the virus (temperature-driven) and the feeding patterns of mosquitoes on humans ended up as other key factors. Including both transmission rate (temperature-driven) and the feeding preference of mosquitoes together with the normalized mosquito numbers improves our predictive power. In effect, the higher virus transmission rates have served to magnify the influence of smaller populations of mosquitos. Calculations of prediction accuracy for a model that was based only on a normalized number of mosquitoes provided an *r* value −0.0675 and RMSE of 0.668 for 2016 data. These goodness-of-fit values are decidedly inferior as compared to calculations coming from a model with three variables represented (*r* = 0.620 and RMSE = 0.541). Thus, it is important to consider both mosquito numbers and transmission factors in modeling the dynamics of disease cases. Moreover, this result supports arguments that larger numbers of mosquitoes do not always lead to a higher risk of infection [7,14]. Table 2 also indicates that predicted disease cases were best correlated to lagged values in normalized numbers of *Culex tarsalis* mosquitoes. Such a result is not surprising given the incubation period of the virus is 3–14 days [63]. Thus, there is a time lag between when the infection would have occurred and when actual symptomatic people would have begun to turn up. These time-lag effects in both key predictive variables explain why the number of disease cases tends to lag the mosquito numbers. This time lag can be beneficial in providing a small window of opportunity to react to potential spikes in WNV incidence. It remains to be seen whether our approach has comparable predictive power. These findings reiterate conclusions from other studies [7,9,10,11] that mosquito abundance is an important factor controlling the numbers of WNV cases. Each season mosquitoes gradually become more infectious with time as the mean temperature increases from spring through summer. The model for human disease cases worked better for times when the observed case numbers were higher. It was less successful in simulating the small number of WNV cases in 2011 (Figure 6B). However, it was always the goal to predict the onset of disease cases, which successfully provided the more virulent peaks in 2012, and 2013.

The selection of model variables for simulating numbers of mosquitoes was somewhat similar to those determined by Chuang et al. [6], and Lebl, Brugger, and Rubel [41]. Chuang et al. [6] used temperature and vegetation opacity, while Lebl, Brugger, and Rubel [41] used day length, temperature, precipitation, relative humidity, and wind velocity. The difference between our model and their models is the inclusion of a non-linear temperature function (mortality rate) rather than raw temperature data. In addition, our model used gage height to examine the water availability for mosquitoes instead of vegetation opacity or precipitation. Our model differed from those in previous studies in the use of raw mosquito numbers instead of a vector index [7,8]. Other variations in the choice of model variables exist as well. For example, Paull et al. [64] used a relative basic reproduction number (mosquito-species specific and temperature-driven) and multiple climate factors to predict the numbers of neuroinvasive disease cases.

The models for mosquito abundance and numbers of WNV cases successfully captured the observed variability. Based on cross-validation results and out-of-sample analyses, the models for mosquito numbers and disease cases possess prediction capabilities for other years. The out-of-sample data analyses for 2016 showed that both models have good predictive performance. The exception is two years (i.e., 2007; 2012) where the model for mosquito numbers exhibited large RMSE values. One possible issue is an inherent limitation in cross-validating data occurring as a relatively short time series. Specifically, in 2012, the patterns in mosquito numbers were somewhat unique as compared to other years. In 2012, the mosquito numbers were unusually small. This pattern was not observed in the other years and probably was more important than other years in characterizing the complete spectrum of behavior in the mosquito population.

The fitness of models from Figure 6A showed good results. The model captured the time associated with peak mosquito numbers and estimated small mosquito numbers during the latter portion of the mosquito season in September. While the model of human disease cases simulated the spillover of disease cases into seasons with relatively small mosquito numbers (i.e., September), differences do exist between the modeling results and actual data. For example, modeled mosquito numbers are larger than measured values for some weeks in 2013. This digression is likely caused by the higher temperatures in those years. The model of human disease cases also failed to estimate the very small number of disease cases observed in 2011. Clearly, other factors, e.g., socio-economic should be considered in future work.

In practice, mosquito control programs vary from county-to-county. Some counties begin spraying when some threshold number is reached for trapped mosquitoes or when WNV is detected in mosquitoes found in traps, or elsewhere. In addition to these practices, our studies here have identified other potential strategies that could be pursued with control strategies. It may be possible to take advantage of the two-week lag in transmission rate. Because jumps in the number of cases follow increases in temperatures, more aggressive spraying could begin in advance of weather forecasts predicting the onset of especially warm weather. Transmission rate drastically increases when the daily mean temperature is above 20 °C. Finally, the mosquito population modeling suggests that years with severe winters provide the risk of greater numbers of mosquitoes. Further studies might consider a more aggressive treatment of waterbodies with larvicides early in the mosquito seasons following specific winter conditions. Public health workers can integrate both models or solely use the human disease case model to predict the risk of WNV two-weeks in advance. However, differentiating the models for mosquitoes and disease cases is important to elucidate the effects of the factors on mosquito numbers and disease cases. This approach will be beneficial to understanding the factors that are directly or indirectly associated to the disease transmission dynamics.

Some limitations of the study data are worth noting. With the mosquito data, unknown influences due to insecticides might produce counting inaccuracies. Studies have found that the efficiency of a light trap can be reduced by the brightness of the moon or nearby exterior lighting (Reinert, 1989). Gaps in the data of collected mosquitoes might introduce bias in the statistical models. Thus, moving the trap locations should be minimized. There is also the question of how many traps are needed to account for the complexity and spatial structure in mosquito environments. Commonly, retrospective studies end up making use of data collected by others. At Bismarck, individual traps are located in areas of ~8 km^2^. These areas are comparable to the sampling areas used in other studies [5,6,58]. Averaging the mosquito numbers weekly from a variety of traps leads to the loss of information concerning population dynamics. In addition, the reported number of human disease cases can be skewed by underreporting of WNV cases. Some people with mild WNV symptoms may seek medical attention and the majority of infected individuals are asymptomatic. Also, errors might arise from presuming that a case reported for Burleigh Country was caused by a mosquito bite in the same location. In this respect, using the mosquito data from the City of Bismarck to predict disease cases across Burleigh County is not a serious problem because 77% of the people in Burleigh County live in [65,66] and around Bismarck. These kinds of unknowable uncertainties in the data can provide uncertainties in the simulation results. Furthermore, our models used temperature-driven functions such as mosquito reproduction rate and the virus transmission rate. These functions were obtained as empirical equations fitted to results obtained from laboratory experiments. However, when we apply these functions to field settings, the temperatures naturally vary over time. Thus, using the mean temperature in the model to determine these functions may not fully capture the dynamics in mosquito numbers or the patterns of virus transmission.

Overall, our models worked well in describing the dynamics of the mosquito population and the human disease cases. They revealed the strength of the association between variables in terms of the mosquito population or human disease cases. However, we cannot totally discount the possibility of bias due to the choice of variables included in the primary models. There is, for example, no assurance that other inherently important factors were neglected. However, we have included variables considered to be important in other studies. Our approach of differentiating factors between mosquitoes and disease cases has the potential for application at a regional scale.

## 5. Conclusions

Two independent GLM models are capable of predicting mosquito abundances and human disease cases, respectively. By partitioning the climatic and transmission factors in a logical manner, it is possible to examine the influences of each factor. These mosquito and human disease models were successful in capturing mosquito abundances and the numbers of disease cases in Bismarck, ND. One important conclusion was that in addition to the normalized number of *Culex tarsalis* mosquitoes, the numbers of human disease cases were strongly associated with the transmission rate of the virus (temperature-driven) and the feeding pattern of mosquitoes. Furthermore, mosquito reproduction rate (temperature-driven), dew points, day length, freeze days, and extreme flooding were shown to be important in affecting the abundance of mosquitoes. There are indications that extensive flooding can produce lags of months to years.

Our findings corroborated conclusions from other work that lags in variables controlling the mosquito numbers and human disease cases can provide a window of opportunity to anticipate a surge in WNV cases. Future studies will be population-based prevention strategies for managing WNV, which will require a more sophisticated understanding of spatial complexities in mosquito distributions, especially in terms of habitats reflected in land-use and land cover.

## Figures and Tables

**Table 1:** Variables used in the mosquito model to predict the weekly mosquito numbers. Coefficients are the parameter estimate of each variable with standard deviation. *p*-Values show the significance of parameters in the model.

Variables ^1^	Coefficients	Std. Error	*p*-Value
intercept	−2.712 × 10^1^	8.696	1.817 × 10^−3^
dew point lag 0	3.980 × 10^−1^	1.025 × 10^−1^	1.041 × 10^−4^
day length lag 2	1.075	5.534 × 10^−1^	5.203 × 10^−2^
freeze days	5.534 × 10^−2^	1.567 × 10^−2^	4.126 × 10^−4^
gage height lag 0	−3.808 × 10^−1^	1.326 × 10^−1^	4.068 × 10^−3^
gage height lag 1 year	−7.062 × 10^−1^	2.258 × 10^−1^	1.765 × 10^−3^
reproduction rate of mosquito lag 0	2.084 × 10^2^	9.733 × 10^1^	3.225 × 10^−2^

^1^ lags of 0, 1, and 2 denotes lag of zero-week, one week, and two weeks.

**Table 2:** Variables used in the WNV case model to predict the weekly human disease cases. Coefficients are the parameter estimate of each variable with standard deviation. p-Values show the significance of parameters in the model.

Variables ^1^	Log Part ^2^			Logit Part ^3^		
	Coefficients	Std. Error	*p*-Value	Coefficients	Std. Error	*p*-Value
intercept	−5.732 × 10^−1^	7.103 × 10^−1^	4.196 × 10^−1^	6.312	2.453	1.009 × 10^−2^
transmission rate lag 2	6.889 × 10^1^	3.078 × 10^1^	2.522 × 10^−2^	3.124 × 10^1^	2.001 × 10^2^	8.759 × 10^−1^
feeding pattern lag 2	−2.297	2.988	4.422 × 10^−1^	−1.110 × 10^−2^	7.440 × 10^1^	1.356 × 10^−1^
mosquito lag 2	1.727 × 10^−1^	1.524 × 10^−1^	2.570 × 10^−1^	−8.143 × 10^−1^	6.195 × 10^−1^	1.887 × 10^−1^

^1^ lags of 0, 1, and 2 denote lag of zero-week, one week, and two weeks; ^2^ Log-linear part denotes when the human disease cases were zero; and ^3^ Logit part expresses the equation when the disease cases were above zero.

## Supplementary Materials

The following are available online at http://www.mdpi.com/1660-4601/15/9/1928/s1: Table S1: Locations of mosquito traps in Bismarck, ND, Table S2: A summary of model variables, ordered according to AICc for the weekly mosquito models, Table S3: A summary of model variables, ordered according to AICc for the models of weekly human disease cases, Table S4: A comparison of RMSE of mosquito model between leave-one-out cross-validation models and the mosquito model that included all year (2007–2015), Table S5: A comparison of RMSE for the model of human disease cases between leave-one-out cross-validation results and the model of human disease cases that included all years (2007–2015), Figure S1: The results of sensitivity analysis with our two statistical models, Figure S2: The state averages are compared to the normalized mosquito number for Bismarck, ND, 2007–2015, Appendix S1: Methods—Ecological factors and transmission factors, Appendix S2: Methods—The generalized linear models, Appendix S3: Mosquito data from regional scale.
