# Supplementary material for: Key Factors Influencing the Incidence of West Nile Virus in Burleigh County, North Dakota
Source: Int J Environ Res Public Health. 2018 Sep 5;15(9):1928. doi: 10.3390/ijerph15091928 (PMC6164257; doi:10.3390/ijerph15091928)
Supplement: Supplementary file 1 [file ijerph-15-01928-s001.pdf]

International Journal of
Environmental Research and Public Health

Key Factors Influencing the Incidence of West Nile Virus in Burleigh County, North Dakota

Hiroko Mori, Joshua Wu, Motomu Ibaraki and Franklin W. Schwartz

Supplementary Material

Appendix S1. Methods - Ecological factors and transmission factors

Equations used to calculate the environmental factors and transmission factors are listed in this Appendix.

The birth rate (b_M) [per day] of mosquitoes is calculated as follows [1]:

$$b_M = 2.325 \times \frac{0.344}{1 + 1.231\, e^{-0.184(T-20)}} \times \frac{1}{10} \tag{1}$$

where T is temperature (°C).

Our equation for the mortality rate (m_M) (per day) of mosquitoes is modified from that of Rubel et al. [1]. The modified equation that follows was better accounted for data of our study area:

$$m_M = 1.414 \times 10^{-6}T^3 + 1.207 \times 10^{-5}T^2 + 1.630 \times 10^{-4}T + 7.7739 \times 10^{-4} \tag{2}$$

Rubel et al. [1] fitted a U shape function to the mosquito mortality rate data obtained from Reisen [2], while we fitted our model to third-degree polynomial function. If m_M took on a negative, we assigned m_M, a value of zero. We preferred the mosquito mortality rate to reach zero at low temperature in order to represent the adult diapause period of mosquitoes [3].

Based on the birth rate and mortality rate of mosquitoes, the reproduction rate (R_M) of mosquitoes was calculated from the subtraction between the mosquito birth rate and mosquito mortality rate.

$$\mathrm{R_M} = \mathrm{b_M} - \mathrm{m_M} \tag{3}$$

The day length (D) in hour calculations are as follows [4]:

$$\mathrm{D} = 24 - \frac{24}{\pi}\left(\cos^{-1}\frac{\sin\frac{\gamma\pi}{180} + \sin\frac{\gamma\pi}{180}\sin\varphi}{\cos\frac{L\pi}{180}\cos\varphi}\right) \tag{4}$$

where γ is day length or 0.8333° for this study, and L is latitude for Bismarck, 46.7825°. The declination angle of the sun (ϕ) is defined as:

$$\varphi = \sin^{-1}(0.39795\cos\theta) \tag{5}$$

where θ, the revolution angle is defined as:

$$\theta = 0.2163108 + 2\tan^{-1}[0.9671396\tan[0.00860(\mathrm{X} - 186)]] \tag{6}$$

where X is the day of the year. The D was also weekly averaged.

The empirical equation for the rate of virus development of mosquitoes (υ) (–) [5] is:

$$\upsilon = -0.132 + 0.0092 \times T \tag{7}$$

where T (°C) is temperature. From this regression line, they determined that the rate of virus development becomes zero at 14.°C.

Mosquito biting rate (ε) (per day) was obtained from a study conducted by Rubel et al. [1]. Their equation is as follows:

$$\varepsilon = \frac{0.344}{1 + 1.231\mathrm{e}^{-0.184(T-20)}}. \tag{8}$$

Kilpatrick et al. [6] collected engorged mosquitoes at field sites and identified its blood meals using microbiological analysis. The preference for mosquitoes to feed on humans gradually increases from May to September following the logistic regression:

$$\beta = \frac{\mathrm{e}^{-7.3+0.025X}}{1 + e^{-7.3+0.025X}} \tag{9}$$

where β is the ratio of mosquito feeding on humans among other animals and X is the numerical value for the day of the year.

Appendix S2. Methods - The generalized linear models

The general equation for generalized liner models (GLMs) is

$$g(E[y]) = a + b_1x_1 + \cdots + b_ix_i \tag{9}$$

where $g()$ is a link function, $E[y]$ is the expected value of the dependent variable, a is an intercept, b is a coefficient, x is a predictive variable, and subscript i is the number of predictive variables [7-9]. For the mosquito model, the dependent variable is numbers of mosquitoes and predictive variables are ecological factors. For the model of human disease cases, the dependent variable is case numbers, determined by predictive variables that include three transmission factors.

Several link functions were selected depending on the mosquito number and human disease case distribution [8,9]. The best model was further chosen between multiple generalized linear regression models using Vuong test [10]. Finally, a negative binomial regression model and a zero-inflated negative binomial regression model were applied for the mosquito model and the human disease cases model, respectively. The negative binomial regression model uses a logarithm link function, λ, given as [11],

$$\lambda = e^{(a+b_1x_1+\cdots+ b_ix_i)}. \tag{10}$$

The expected value in mosquito numbers is calculated as:

$$E[mosquito] = \lambda \tag{11}$$

while a zero-inflated negative binomial regression model consists of logistic (ρ) and logarithmic (λ) link functions. The expected human disease cases $E[human\ disease\ cases]$ are derived as follows,

$$E[human\ disease\ cases] = (1-\rho)\lambda \tag{12}$$

where the logistic link function, ρ represents the probability of having a zero and the logarithmic link function, λ predicted numbers of expected human disease cases. The logistic link function (ρ) is defined as:

$$\rho = \frac{1}{1 + e^{-(a+b_1x_1+\cdots+ b_ix_i)}}. \tag{13}$$

Values for the intercept a and coefficient b are assigned individually for logistic and logarithm link functions.

Appendix S3: Mosquito Data from Regional Scale

Figure S2 shows the yearly normalized mosquito numbers from 49 sites. The variability in normalized mosquito numbers was generally small among the average values for the 49 sites in North Dakota and Bismarck. This result suggests that the small mosquito numbers for 2012 were not an anomaly of trap counts in Bismarck. Therefore, we assumed that the mosquito data from Bismarck characterizes the mosquito dynamics and is plausible to use for our local scale analysis.

Table S1. Locations of mosquito traps in Bismarck, North Dakota. The trap locations listed are also depicted on Figure 1. The data for 2007 were collected from two trap locations because data were not available from the Environmental Health Division for the City of Bismarck but obtained from the North Dakota Department of Health.

Trap location	2007	2008	2009	2010	2011	2012	2013	2014	2015	2016	Latitude	Longitude
1											46.81561	-100.79248
2											46.79467	-100.80171
3											46.78439	-100.81364
4											46.83273	-100.81842
5											46.83297	-100.76106
6											46.82893	-100.75385
7											46.81126	-100.74323
8											46.80623	-100.73235
9											46.79459	-100.77924
10											46.79578	-100.77229
11											46.78623	-100.78813
12											46.77998	-100.77361
13											46.85546	-100.80178
14											46.85172	-100.77499
15											46.89578	-100.84431
16											46.84332	-100.71913
17											46.84799	-100.71959
18											46.83489	-100.76102

Table S2 A summary of model variables, ordered according to AICc for the weekly mosquito models. Each row shows the coefficients determined by the model as the best fit. The first row provides the best model coefficients used to predict the numbers of mosquitoes. Only the top 10 best-fitted models are listed in the table. No parameter numbers indicate that those variables were not included in the fitting model.

variables										
intercept	dew point lag 0	day length lag 2	freeze days	gage lag 0	gage lag 1 year	precipitation lag 0	reproduction rate lag 0	snow	wind velocity lag 2	AICc
-27.12	0.3980	1.075	0.05534	-0.3808	-0.7062		208.4			649.1
-11.83	0.3734		0.05916	-0.4387	-0.6998		286.9			649.6
-31.54	0.3942	1.328	0.05577	-0.3979	-0.7182	7.649×10^{-3}	237.8			650.6
-31.70	0.5351	1.554	0.04581	-0.4009	-0.7765					651.0
-27.59	0.4183	1.065	0.05431	-0.3792	-0.7307		206.3		0.1433	651.0
-27.03	0.3991	1.073	0.05323	-0.4177	-0.6774		208.9	1.549×10^{-4}		651.4
-12.48	0.3918		0.05881	-0.4367	-0.7152		283.4		0.1345	651.4
-11.75	0.3748		0.05672	-0.4787	-0.6682		287.7	1.722×10^{-4}		651.7
-12.05	0.3703		0.05952	-0.4468	-0.7021	2.103×10^{-3}	300.2			651.9
-31.44	0.4115	1.289	0.05480	-0.3958	-0.7382	7.088×10^{-3}	234.2		0.1245	652.7

Table S3 A summary of model variables, ordered according to AICc for the models of weekly human disease cases. Each row shows the coefficients determined by the model as the best fit. The first row provides the best model coefficients used to predict the human disease cases. Since the model uses only three variables, all combination of models is listed below. No parameter numbers indicate that those variables were not included in the fitting model.

variables								
Log part				Logit part				
intercept	transmission rate lag 2	feeding pattern lag 2	mosquito lag 2	intercept	transmission rate lag 2	feeding pattern lag 2	mosquito lag 2	AICc
-0.5732	68.89	-2.297	0.1727	6.312	31.24	-111.0	-0.8143	310.9
-0.7218	82.31	-1.779		4.496	-100.3	-59.43		310.9
0.6996		-5.538	0.3317	6.598		-105.3	-0.7347	311.6
0.7552		-4.926		5.612		-92.56		316.5
-0.4588	74.47			1.600	-136.4			318.2
-0.3869	67.07		0.1036	1.633	-128.8		-0.1292	321.0
0.2080			0.2337	0.327			-0.3417	345.7
0.2384				0.109				349.1

Table S4. A comparison of root mean square error (RMSE) of mosquito model between leave-one-out cross-validation models and the mosquito model that included all year (2007-2015).

Year	Cross-validation RMSE	Full year model RMSE
2007	149.2	107.9
2008	31.74	23.54
2009	36.61	29.09
2010	34.88	27.78
2011	29.90	23.28
2012	5412	0.5738
2013	402.0	181.7
2014	51.65	37.80
2015	20.07	15.85

Table S5. A comparison of RMSE for the model of human disease cases between leave-one-out cross-validation results and the model of human disease cases that included all years (2007-2015).

Year	Cross-validation RMSE	Full year model RMSE
2007	2.737	2.838
2008	0.7798	0.7399
2009	0.5625	0.5576
2010	0.7259	0.6740
2011	0.6995	0.6587
2012	0.8267	0.7952
2013	1.276	1.335
2014	0.5298	0.5419
2015	0.8278	0.7268

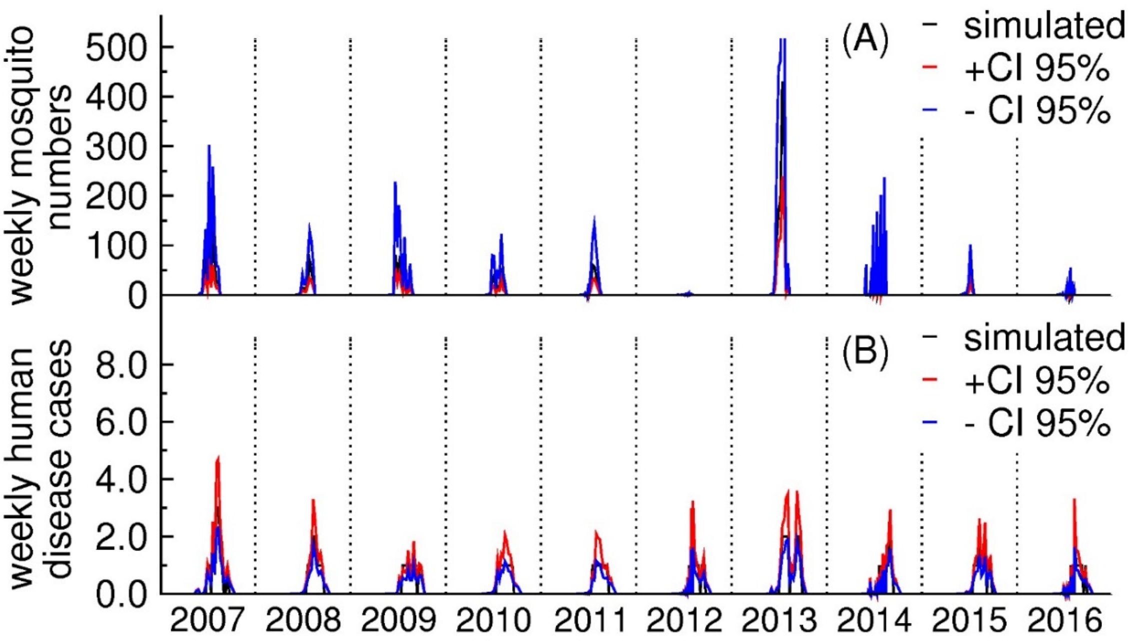

Figure S1. The results of sensitivity analysis with our two statistical models. The mosquito reproduction rate and the virus transmission rate are adjusted from the best fit model results to upper and lower bound of the 95% Confidence Interval (CI). The weekly estimates of mosquito numbers were most sensitive to variations in the reproduction rate of mosquitoes. The predicted numbers of human disease cases were much less sensitive to variations in the virus transmission rate.

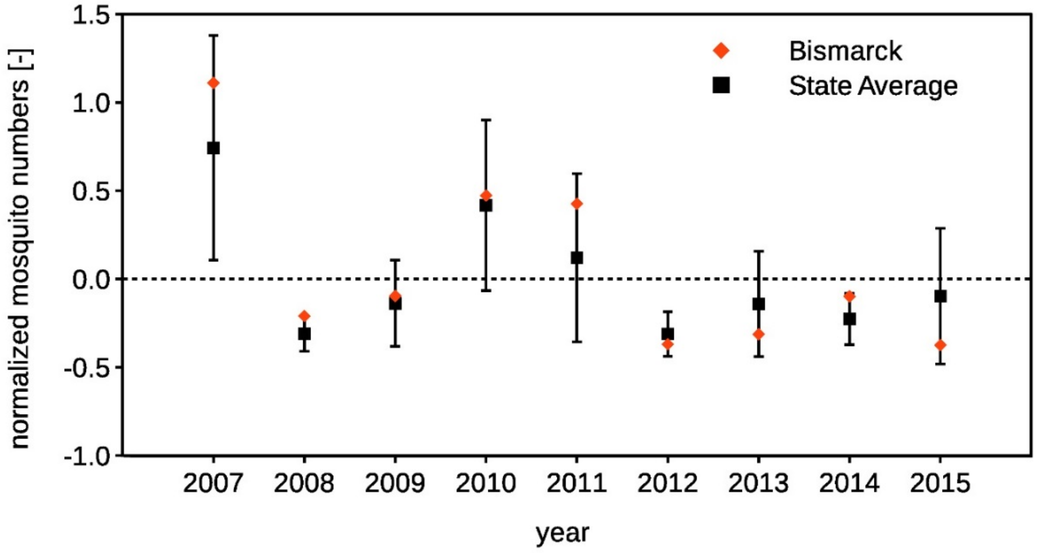

Figure S2. The state averages are compared to the normalized mosquito numbers for Bismarck, North Dakota, 2007 – 2015. The state average is averages of normalized data for 49 sites in North Dakota. The black bar represents the standard deviation of normalized mosquito numbers in 49 sites.

References

1. Rubel, F.; Brugger, K.; Hantel, M.; Chvala-Mannsberger, S.; Bakonyi, T.; Weissenböck, H.; Nowotny, N. Explaining Usutu Virus Dynamics in Austria: Model Development and Calibration. *Prev. Vet. Med.* **2008**, *85*, 166-186.
2. Reisen, W.K. Effect of Temperature on Culex Tarsalis (Diptera: Culicidae) from the Coachella and San Joaquin Valleys of California. *J. Med. Entomol.* **1995**, *32*, 636-645.
3. Denlinger, D.L.; Armbruster, P.A. Mosquito Diapause. *Annu. Rev. Entomol.* **2014**, *59*, 73-93.
4. Forsythe, W.C.; Rykiel, E.J.; Stahl, R.S.; Wu, H.; Schoolfield, R.M. A Model Comparison for Daylength as a Function of Latitude and Day of Year. *Ecol. Model.* **1995**, *80*, 87-95.
5. Reisen, W.K.; Fang, Y.; Martinez, V.M. Effects of Temperature on the Transmission of West Nile Virus by Culex Tarsalis (Diptera: Culicidae). *J. Med. Entomol.* **2006**, *43*, 309-317.
6. Kilpatrick, A.M.; Kramer, L.D.; Jones, M.J.; Marra, P.P.; Daszak, P. West Nile Virus Epidemics in North America are Driven by Shifts in Mosquito Feeding Behavior. *PLoS Biol* **2006**, *4*, e82.
7. Nelder, J.A.; Wedderburn, R.W.M. Generalized Linear Models. *Journal of the Royal Statistical Society.Series A (General)* **1972**, *135*, 370-384.
8. McCullagh, P.; Nelder, J.A. *Generalized Linear Models*; Chapman and Hall: London, 1983; pp. 15-34.
9. Madsen, H.; Thyregod, P. *Introduction to General and Generalized Linear Models*; CRC Press, 2010; pp. 1-8, 87-156.
10. Vuong, Q.H. Likelihood Ratio Tests for Model Selection and Non-Nested Hypotheses. *Econometrica* **1989**, *57*, 307-333.
11. Hilbe, J.M. *Modeling Count Data*; Cambridge University Press: New York, USA, 2011; pp. 1-161, 172-209.

© 2018 by the authors. Submitted for possible open access publication under the terms and conditions of the Creative Commons Attribution (CC BY) license (http://creativecommons.org/licenses/by/4.0/).
